# New Classification System and Defect-Oriented Algorithm for Functional Soft-Palate Reconstruction with Buccinator Myomucosal Flaps

**DOI:** 10.3390/jcm13247766

**Published:** 2024-12-19

**Authors:** Olindo Massarelli, Lisa Catarzi, Guido Gabriele, Flavia Cascino, Andrea Frosolini, Paolo Gennaro

**Affiliations:** Department of Maxillofacial Surgery, University of Siena, 53100 Siena, Italy; molindo74@gmail.com (O.M.); guido.gabriele@unisi.it (G.G.); flavia.cascino@ao-siena.toscana.it (F.C.); frosolini@student.unisi.it (A.F.); paolo.gennaro@unisi.it (P.G.)

**Keywords:** oropharyngeal reconstruction, soft-palate reconstruction, oropharyngeal defect, buccinator myomucosal flap, cheek myomucosal flap, facial artery flap, FAMM, velopharyngeal insufficiency

## Abstract

**Background/Objectives**: Currently, there is a lack of a comprehensive classification system for soft-palate defects that provides synthetic information to guide functional reconstructive treatment. Our awareness, shaped by extensive experience, of the superiority of myomucosal flaps to fasciocutaneous flaps in functional palate reconstruction has driven us to introduce a new defect-based classification system and propose a new algorithm for reconstructing soft-palate defects using buccinator myomucosal flaps. **Methods**: Soft-palate defects were classified into five classes. A reconstruction algorithm employing buccinator myomucosal flaps—including axial, island, and tunnelized flaps along with their variants as described in previous studies—was utilized. Clinical records, including tumor stage, location, defect size, and details of the myomucosal flap used, were documented. Postoperative speech intelligibility, swallowing, and quality of life (QoL) were evaluated. Donor-site morbidity and complications were also assessed. Spearman’s rank correlation was employed to assess relationships between clinical parameters and functional outcomes. **Results**: Twenty-two patients who had undergone soft-palate resection and subsequent reconstruction were reviewed. Favorable recovery of swallowing and speech was reported in all cases, with a median deglutition score of 6.04 ± 0.85 and no severe velopharyngeal insufficiency observed (speech score: 0.36 ± 0.58). Quality of life assessments indicated satisfactory recovery across physical, social, emotional, and functional parameters. Donor-site morbidity was low (average score: 8.3), with only minor complications observed. Tumor stage showed a significant correlation with speech score (r = 0.44, *p* = 0.04). **Conclusions**: The proposed classification introduces a comprehensive, simple, and user-friendly categorization of soft-palate defects, accompanied by a myomucosal reconstructive algorithm designed to guide surgeons through the reconstructive process, aiming to provide optimal functional reconstruction. The study’s small sample size and monocentric design may have limited the detection of meaningful correlations, highlighting the need for larger, multicentric studies with objective methods to validate findings.

## 1. Introduction

The soft palate is an unpaired structure that spans the midline and defines both the roof of the oropharynx and the floor of the nasopharynx. It is the most important component of the velopharyngeal mechanism, which also includes the posterior and lateral pharyngeal walls. This organ is responsible for proper speech production and resonance and is intimately associated with the complex functions of swallowing and respiration. Functional deficits are not uncommon after resection of tumors of the soft palate, which can result in velopharyngeal incompetence that strongly affects the patient’s quality of life. Furthermore, the severity of the defect and the reconstruction method can have significant postoperative functional repercussions [1].

Currently, several classification systems categorize defects in the oropharynx and soft palate [1,2,3,4]. Among them, the recent ones are all based on defects created by transoral transrobotic surgery (TORS) [3,4]. However, the open approach appears to still be a very common approach for soft-palate tumor resection.

The limitations of these classification systems lie in their primary focus on solely lateral oropharyngeal wall defect or isolated median soft-palate defect sparing lateral pharyngeal arch, thereby disallowing a comprehensive description of all possible kinds of defects encountered in current clinical practice. Moreover, they fail to provide information that would easily guide reconstructive treatment, which poses a difficult surgical challenge in the soft-palate region. The main goal of the reconstruction is to simulate the function of the soft palate whenever possible [5]. Closure of oroantral communication and restoration of a functional myomucosal velum should be the principal concerns. So far, numerous procedures and flaps have been proposed to reconstruct the soft-palate region. However, the majority of publications merely outline the commonly used flaps by each surgeon, lacking a comprehensive assessment of functionality [2,5].

Traditionally, surgical techniques for soft-palate reconstruction have focused on restoring the morphology of its two layers (oral and nasal), as this was believed to yield the best functional outcomes. However, clinical results suggest that a volumetrically perfect anatomical restoration does not always result in optimal functional outcomes. Kimata [1] argued that the primary soft-palate reconstructive goal should be to narrow the residual velopharyngeal space, which, as Penfold [6] emphasized, is the most critical factor in determining functionality. A residual velopharyngeal space of 0.4 cm^2^ is required for good speech intelligibility and effective swallowing. Exceeding this parameter can result in hypernasality and regurgitation during swallowing, while smaller values can lead to impaired nasal breathing or even sleep apnea.

Over the decades, various combinations of local mucosal flaps have been described for reconstructing the soft palate, including pharyngeal flap superiorly or inferiorly based, the pharyngeal flap combined with a palatal flap, pedicled buccal fat pad flap, and pedicled intraoral cheek transpositions [5,7,8,9].

These methods have been reported to effectively restore swallowing and speech functions with minimal operative effort. However, the use of two (or three) local flaps appears to be complex and may lead to significant oral cavity morbidity.

The selection of the flap type should ensure the restoration of the lost form of the organ while maintaining its potential functionality with the simplest procedure. Indeed, an “ideal reconstruction” is achieved using the same or a similar kind of tissue. The myomucosal flaps adhere to this fundamental surgery plastic axiom in the soft-palate reconstruction [10].

Following this principle, in the present study, we introduce a new classification system for soft-palate defects and an associated reconstructive algorithm using buccinator myomucosal flaps, both grounded in our experience with reconstructing soft-palate defects after oncologic surgery. As a secondary aim, the study explores the relationships between clinical factors (e.g., T stage, flap size) and functional outcomes (speech, deglutition, and quality of life) through correlation analysis, to evaluate the applicability of the proposed system while generating insights into factors influencing postoperative recovery.

## 2. Materials and Methods

### 2.1. Study Design

A retrospective study was conducted involving patients with soft-palate post-ablative defects who underwent primary resection and reconstructive surgery with buccinator myomucosal flaps between January 2008 and July 2024 in a tertiary referral hospital. Patients were excluded from the present study if (1) surgical resection encompassed any structures within the oral cavity, notably the oral tongue and or/maxilla, or (2) the reconstruction method diverged from the subsequent descriptions. The study protocol was designed in conformity with the ethical guidelines of the 1964 Declaration of Helsinki and was approved by the Ethical Committee for Clinical Research of the University Hospital of Siena (approval no. 13/2024, 21 June 2024).

### 2.2. Classification Proposal

**Figure 1:**
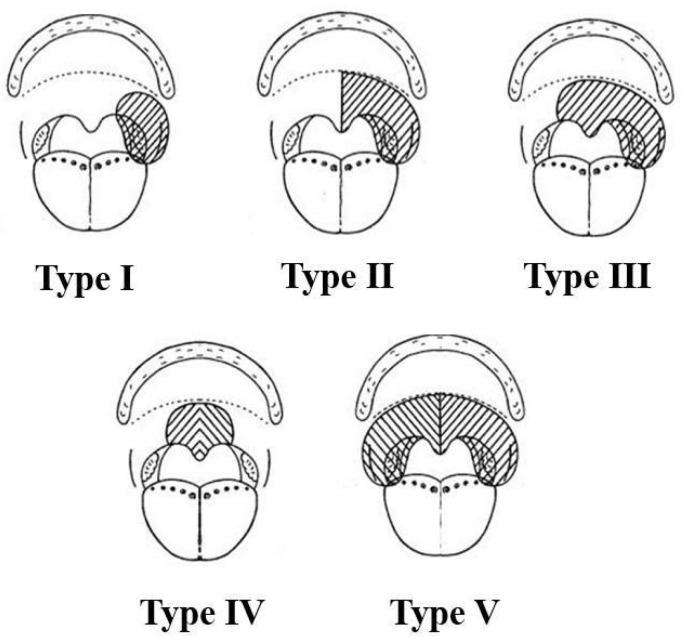
Classification system. The diagonal shading indicates the defect. Type I: defect located in the lateral oropharyngeal wall. Type II: defect extends to the superior oropharyngeal wall. Type III: defect extends to the contralateral side. Type IV: pure middle soft-palate defect sparing the lateral thirds of the soft palate. Type V: defect extends to total soft palate and bilateral palatopharyngeal arch.

- The authors classified soft-palate defects into the following types (Figure 1):
- Type I: localized in the lateral oropharyngeal wall and including palatopharyngeal, palatoglossal, and superior pharyngeal constrictor muscles.
- Type II: localized in the hemisoft palate including its muscles (tensor veli palatini and levator veli palatini muscles) and extending to the homolateral lateral oropharyngeal wall (palatopharyngeal, palatoglossal, and superior pharyngeal constrictor muscles), named as palatopharyngeal arch. This resection can include, as required, the ipsilateral half of the uvula excision.
- Type III: the same defect as Type II extending to the contralateral soft palate and its muscles, sparing the contralateral palatopharyngeal arch.
- Type IV: a pure middle soft-palate defect involving the uvula including the uvulae muscles, medial third of palatine aponeurosis, and sparing the lateral thirds of the soft palate.
- Type V: involves the whole soft palate and bilateral palatopharyngeal arch, including its muscles (i.e., bilateral tonsillar fossae).

This new classification system aims to provide a simple and practical tool to support a decision-making algorithm for soft-palate reconstruction.

### 2.3. Reconstructive Algorithm and Operative Technique

**Figure 2:**
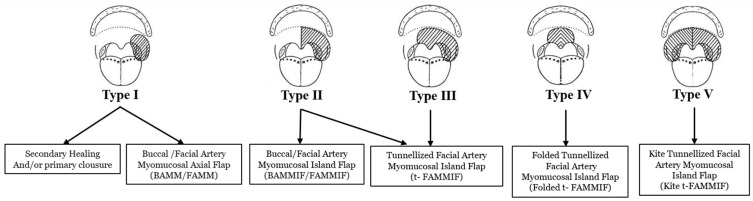
Defect-oriented algorithm for functional soft palate reconstruction.

The reconstructive algorithm we used is depicted in Figure 2. In general, using this classification system, defects were divided into one of five classes with increasing complexity and increasing need for more advanced reconstructive procedures.

In Type I, defects were typically reconstructed with secondary healing, primary closure, or local axial myomucosal flaps such as pedicled facial artery myomucosal (FAMM) flap and buccal artery myomucosal (BAMM) flap [11]. The FAMM flap is a flap centered on the course of the facial artery with an orthograde flow (inferiorly based) or a reverse flow (superiorly based) [12], also classified as nasal artery myomucosal (NAMM) flap [12]. The BAMM flap is a flap pedicled on the buccal artery, with a posterior mucosal pivot at the pterigomandibular raphe with a horizontal axis on the oral commissure [13].

In Type II, when defects involved half of the entire soft palate, myomucosal island flaps were always needed to reach the defect. The facial artery myomucosal island flap (FAMMIF) allowed the rising of almost all the cheek mucosa as an island flap based on the facial artery angiosome but also included the adjacent buccal artery angiosome, to augment peripheral vascularization [14]. Otherwise, in the buccal myomucosal island flap (BAMMIF), the facial artery was ligated but harvested with the flap to maintain the integrity of the cheek vascular network. Arterial blood flow is assured by the buccal artery, which is meticulously dissected up to the pterygomandibular raphe to increase its arc of rotation. Furthermore, in selected cases, as dentate patients, the tunnelized facial artery myomucosal island flap (t-FAMMIF) was employed. This represents a technical advancement, as previously detailed by Massarelli et al. [11], exploiting all the reconstructive features of this flap. With t-FAMMIF, facial vessels are dissected up to their origin in the neck. The flap is carried out to the neck region, through a short paramandibular tunnel created on the inferior fornix following the course of the vessels. The flap is then re-routed through the anterior tonsillar pillar utilizing another tunnel created medially to the mandible.

Reconstruction of Type III typically requires reconstructions with the tunnelized buccinator myomucosal island flap (t-FAMMIF), which increases the flap’s rotation arc and allows for the reconstruction of the contralateral palatal site.

**Figure 3:**
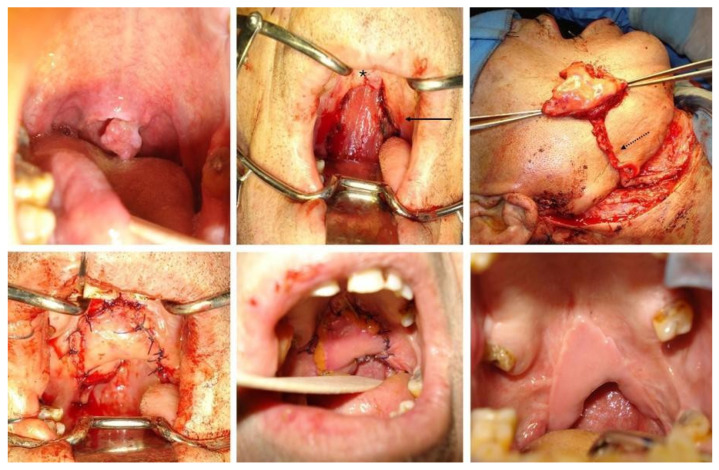
Clinical case of squamous cell carcinoma of the soft palate centered on the uvula (T2N0M0). The soft-palate defect is classified as Type IV and reconstructed with a folded tunnelized facial artery myomucosal island flap (Foded t-FAMMIF). The arrow indicates palatopharyngeal, palatoglossal, and superior pharyngeal constrictor muscle areas, which are spared. The asterisk represents the hard palate. The dotted arrow indicates the flap pedicle (facial vessels).

The Type IV defects, which involve both (nasal and oral) layers of median soft palate, were reconstructed with a tunnelized facial artery myomucosal island flap harvested in a “folded fashion” (Folded t-FAMMIF) [15]. It contains sufficient tissue for the reconstruction of full-thickness soft-palate defect while avoiding scar retraction and palatal dehiscence. The flap reached the soft-palate defect by routing the pedicle through another tunnel created in the right anterior pillar of the tonsillar fossa, medially to the mandible, and the flap was then sutured to the residual palate (Figure 3).

**Figure 4:**
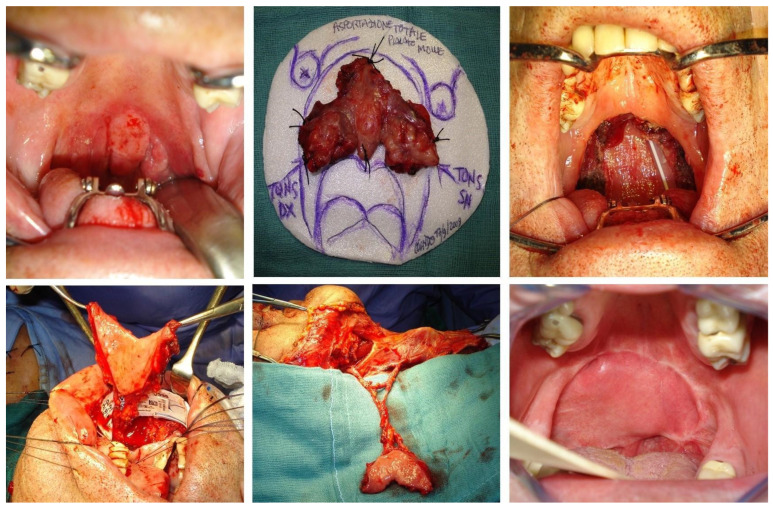
Clinical case of squamous cell carcinoma of the soft palate (T3N0M0). The soft-palate defect is classified as Type V and reconstructed with Kite tunnelized facial artery myomucosal Island Flap (Kite t-FAMMIF).

For the Type V defects, the largest defect of our classification, a single unfolded “kite-shaped” tunnelized facial artery myomucosal island flap (Kite-shaped t-FAMMIF) [16] was employed. This flap was harvested as previously described, but “parachuted” over the soft-palate defect as a kite, with the mucosa toward the oral cavity and the buccinator muscle toward the rhinopharynx [16]. Therefore, the mucosal and muscular sides of a single t-FAMMIF can restore the oral and nasal lining of the palate, respectively. The flap was sutured as a “tent”, in slight tension from the posterior edge of the hard palate and to bilateral residual tonsillar fossae, properly narrowing the velopharyngeal posterior space (Figure 4). In all kinds of flaps, the cheek donor-site defect was repaired by advancing a pedicled buccal fat pad flap, which was spontaneously covered with mucosa.

All cases were treated by the same surgeon (O.M.), who has over 20 years of experience in oral reconstruction.

### 2.4. Postoperative Assessment and Functional Analysis

Outcome data were extracted from the patient’s clinical records, encompassing details such as tumor type, location, and dimensions, as well as the type and size of the myomucosal flap used, harvesting time, and any postoperative complications at both the recipient and donor sites. Each case was evaluated by a multidisciplinary team consisting of a head and neck surgeon, a speech therapist, and a psychologist. At 12 months after surgery, speech intelligibility, deglutition, and quality of life (QoL) were evaluated, after the surgery or upon completion of any adjuvant therapy, if performed.

Speech intelligibility with a 4-point scoring system was assessed as follows: velopharyngeal function (VPF) “0”: competent, “1”: borderline competent, “2”: mild-to-moderate velopharyngeal insufficiency (VPI), and “3”: severe VPI. Speech samples included target consonants embedded in single words, connected speech, and nasal syllable chains. These were recorded using standardized methods to ensure consistency. Results were quantified by evaluating errors in consonant production, hypernasality, and velopharyngeal function, as described by Lohmander A. et al. [17].

Deglutition was evaluated objectively using a method described previously by Teichgraeber et al. [18] with a 7-point scoring system. The score could range from 1 (severe complaints and unable to swallow) to 7 (no complaints). The patient received foods with four different consistencies: liquid, semiliquid, gelatinous, and solid. A quantity equivalent to a spoonful was provided for each consistency. Following this, the patient was instructed to perform a swallowing action. The ability to swallow effectively was assessed by examining the amount of food residue left on the palate after the swallowing action. The overall score was determined by calculating the mean of the scores for the four foods administered.

Patients were given a questionnaire, the Functional Assessment of Cancer Therapy—Head and Neck Questionnaire [19], asking about their QoL. Donor-site morbidity was assessed by evaluating mouth opening, oral commissure symmetry, mucosal lining symmetry of the cheek inner vestibule restoration, and the resulting overall esthetic quality. Each parameter was given a score of 0 to 3, with the scores summed to obtain an overall assessment. Postoperative complications were also registered [20].

### 2.5. Statistical Analysis

A Cohen’s d = 0.5 effect size was used for sample size calculation, reflecting anticipated strong associations between clinical factors (e.g., T stage, flap size) and functional outcomes (speech, deglutition) [21,22]. With a significance level of α = 0.05 and power of 80%, the required sample size was estimated at 23 patients. A power level of 80% is widely accepted as a threshold in clinical and exploratory research as this level balances the ability to detect meaningful effects with the feasibility of achieving the required sample size [22]. Categorical variables were reported as frequencies and percentages, while continuous variables were expressed as mean and standard deviation (SD). Spearman’s rank correlation was employed to assess relationships between clinical parameters (T stage, age, defect type, flap size) and functional outcomes (speech, deglutition, and quality of life scores). This non-parametric method is robust for ordinal and non-normally distributed data [23]. A significance threshold of *p* < 0.05 was used to identify potential trends in this exploratory study. Correlations were categorized as small (r = 0.1−0.3), moderate (r = 0.3−0.5), or large (r > 0.5) [24]. Sample size calculations were performed using G*Power (Erdfelder, Faul, & Buchner, 1996; version 3.1.9.6), while statistical analyses were conducted using SPSS software (Windows, version 12.0; SPSS Inc., Chicago, IL, USA) [21,22].

## 3. Results

In the past decade, we have employed buccinator myomucosal flaps in 103 oral and oropharyngeal surgical reconstructions. Among them, 22 flaps were used for the reconstruction of soft-palate defects after oropharyngeal squamous cell carcinoma removal and included in the present study.

A total of 22 patients, 16 (72.7%) male and 6 (27.3%) female, who had a mean age of 65.4 (SD 8.4 yr; range 54 to 83 yr), underwent soft-palate reconstruction using buccinator myomucosal flaps from January 2008 to January 2024.

Patients were classified as T1 in 3 (13.6%) cases, T2 in 10 (45.5%) cases, T3 in 8 (36.3%) cases, and T4 in 1 (4.6%) case. Tumor resection was coupled with lymph node neck dissection based on oncological guidelines. In 18 (81.8%) patients with cN0 staging, the preservation of facial vessels during neck dissection allowed for the harvesting of the buccinator myomucosal island flap based on the facial vessels. In 2 (9.1%) out of 4 cases staged as N2, flaps were derived from the buccal artery, while in the remaining 2 (9.1%), flaps were islanded on the contralateral facial artery.

Defects were classified as Type I in 4 (18.2%) cases, Type II in 8 (36.4%) cases, Type III in 3 (13.6%) cases, Type IV in 3 (13.6%) cases and Type V in 4 (18.2%) cases. In all cases, the proposed algorithm was followed. The mean flap size was 28.2 ± 8, and the mean time of harvesting the flap was 46.7 ± 5.7 min. Patient demographics, the categorization of defects, and the type of flap utilized are summarized in Table 1.

During the follow-up, no complete flap losses or major complications were reported. Three minor complications were identified: one case of venous stasis was resolved spontaneously, and two cases exhibited marginal necrosis with minor dehiscence of the surgical wound. Donor-site morbidity was very low; the average donor-site morbidity score was 8.3.

Most patients reported minimal complaints related to swallowing and could swallow without difficulty. The median deglutition score was 6.04 ± 0.85. No patient developed severe velopharyngeal insufficiency: speech score was 0.36 ± 0.58 (Table 2). Quality of life assessment revealed a satisfactory recovery of physical, social, emotional, and functional parameters (Table 3).

The correlation analysis revealed one significant relationship between independent variables and functional outcomes: T stage showed a moderate positive correlation with speech score (rs = 0.44, *p* = 0.04), indicating that advanced tumor stages were associated with poorer speech outcomes. Age showed a trend toward significance with deglutition score (rs = 0.37, rs = 0.37, rs = 0.37, *p* = 0.09, *p* = 0.09, *p* = 0.09), indicating a potential age-related effect on swallowing function. However, no significant correlations were found between type of defect or flap area and the seven dependent variables, as detailed in Table 4.

## 4. Discussion

Our classification aims to fill the currently existing gap in the evaluation of soft-palate defects resulting from tumor excision and to guide reconstruction in a simple and intuitive way.

The soft palate is a subsite of the oropharynx, and when affected by a tumor, often locally aggressive squamous cell carcinoma, can face significant functional morbidities, including difficulties with speech, mastication, and swallowing.

Size, location, and contiguous spread of the primary tumor are important factors in the prognosis. Extension outside the palatine arch adversely affects patient survival. Patients with midline tumors and those that extend across the palatine arch have poorer survival rates, because of a higher incidence of regional metastasis.

The dynamic nature of the soft palate makes it a crucial structure in determining velopharyngeal continence, which is difficult to restore functionally, and the size and location of defects affect its outcome with possible detriment to patients’ quality of life. For these reasons, various classifications have tried to create a common and simple language to define these defects. Despite the efforts of many authors, the current main classification systems of soft-palate defects do not classify all types of soft-palate subsites, but only a part of them. All classification systems described to date focus on defects that predominantly affect the lateral wall of the pharynx or only the central portions of the palate, but not those defects that extend to affect both pharyngeal walls bilaterally. Furthermore, the most recent ones are based on TORS and therefore cannot be used to describe defects that follow an open approach. Finally, no classification has, so far, led to a simple and effective reconstructive algorithm that can be applied in daily practice. Almeida et al. [4] proposed a classification system with four classes including seven different kinds of oropharyngeal defects, based on sites and number of subsites involved, internal carotid artery exposure, and pharyngocervical communication with the neck, derived from their experience in reconstructing oropharyngeal defects after TORS.

Jurado et al. [2], starting from the standard anatomical classification of the oropharynx into three main areas—the soft palate, the base of the tongue, and the lateral oropharyngeal wall—categorized the surgical defects of each area into three classes, resulting in a total of nine distinct types of defects.

Despite their efforts, Almeida et al. [4] and Jurado et al. [2] provided extremely complex classifications that do not allow an immediate understanding of the type of reconstruction to be performed in relation to the defect, which is useful for comparing functional results.

Recently, De Virgilio et al. [3] focused their classification only on squamous cellular carcinoma arising in the lateral oropharynx treated exclusively with TORS. This classification, although very detailed, has limited use for the robotic transoral approach. In 2002, Kimata proposed a simple and intuitive classification of lateral and superior oropharyngeal defects, describing three classes of defect: Type I which is located in the lateral oropharyngeal wall; Type II where the defect extends to the superior oropharyngeal wall; and Type III where the defect extends to the contralateral side [1]. This simplified classification of oropharyngeal defects has the merit of providing a useful tool to describe some of the most frequent defects of the soft palate, which can help the surgeon choose the best reconstructive method for that type of oropharyngeal defect.

However, this classification basically focuses on the lateral pharyngeal walls, neglecting central defects or complete ablation of the soft palate, and does not provide an exhaustive description of all possible types of post-ablative defects found in current clinical practice.

In 2024, Matsumoto, Kimata et al. described three additional types of soft-palate defects, focused on the midpoint of the soft palate [24]. However, even in this subsequent article, he did not offer a comprehensive systematization of soft-palate defects or propose a uniform and straightforward reconstructive strategy. Notably, he only addressed central defects of partial or full thickness, without including the lateral pharyngeal arch, which is frequently a site of tumor spread.

Furthermore, these defects were addressed to be reconstructed using either a pharyngeal flap alone or in combination with a free fasciocutaneous flap. However, the pharyngeal flap offers a limited amount of tissue and often needs to be harvested alongside a free flap, leading to more complex surgery and increased donor-site morbidity.

Striving to overcome the shortcomings of these classification systems, the authors developed a new, simple, and comprehensive classification system for current soft-palate defects, providing a practical reconstructive algorithm using only one type of flap, derived from their extensive surgical experience with buccinator myomucosal flaps. The novelty of this study lies in its presentation of a complete classification system for soft-palate defects. It retains the three defect types first described by Kimata in 2002 and adds two new, well-defined types: Type IV, a pure median soft-palate defect centered on the uvula, sparing the lateral pharyngeal arches; and Type V, a defect involving the loss of the entire soft palate, extending bilaterally to affect both tonsillar fossae. This classification system accounts for every possible soft-palate and lateral oropharyngeal defect currently encountered by surgeons in clinical practice, numbering them progressively with five Roman numerals. Each type is clearly described by the extent of palatal excision performed. The extent of soft-palate involvement warrants great consideration for the reconstructive surgeon. Several studies have described poor swallowing and speech intelligibility, as well as higher rates of aspiration associated with increasing size of the defect [4,25,26,27]. Our proposed classification is based on the increasing transverse extent of the soft-palate defect, which is one of the most significant predictors of adverse outcomes. Additionally, it recommends functionally effective local reconstruction using the myomucosal flap and its variants for all types of defects. In our previous reports, we have already demonstrated the effectiveness of buccinator myomucosal flaps in soft-palate reconstruction, supported by optimal functional test results [10,25]. These flaps are versatile and meet most criteria for ideal like-with-like soft tissue reconstruction in the oral cavity and oropharynx [28]. While buccinator myomucosal flaps are commonly used for palate reconstruction, they are generally reserved for smaller defects. However, the use of tunnelized or island variants of this flap can overcome these limitations, enabling the reconstruction of more distant areas and/or larger defects [29]. Buccinator myomucosal flaps are based on the anastomotic network between the facial and buccal arteries. Historically, their use was limited to defects located near the donor site. However, with the advent of the tunneling technique, it is now possible to significantly expand the flap’s rotation arc and, most importantly, eliminate the need for a second surgical stage for pedicle division [11,15,16,29]. The oncological safety of preserving facial vessels during selective dissection has been previously demonstrated for myomucosal flaps [30]. Moreover, buccinator myomucosal flaps can still be safely harvested using the buccal vessels (BAMMIF), even in patients classified as cN+ or in cases where the facial artery was ligated or accidentally severed [11]. In this study, we have demonstrated the feasibility of using buccinator myomucosal flaps to reconstruct all palate defects, including the most complex ones (Types III–V), two of which—Types IV and V—are described here for the first time in a classification system.

Since 2013, Massarelli et al. [15] believed that myomucosal flaps can provide the best functional results, and first described the double-layer reconstruction of a soft-palate defect using a single “folded” t-FAMMIF. Indeed, they noted a certain degree of widening and thinning of the flap over time, which is desirable to minimize the risk of velopharyngeal insufficiency. In a Type IV defect, the edges of this type of myomucosal flap align well with the dissected edges of the recipient site, both on the nasal and oral sides. This alignment provides an optimal contact surface, effectively preventing velopharyngeal dehiscence and postoperative insufficiency.

It is well known that extensive soft-palate resection, such as in Type V defects, can lead to velopharyngeal insufficiency, hypernasal speech, and reduced speech intelligibility. In our cases, the ‘Kyte flap’ effectively restored the oral and nasal lining of the soft palate using the mucosal and muscular sides of a single t-FAMMIF. This technique allows for the harvesting and safe transfer of nearly all the cheek mucosa without the need for folding, as mucosalization of the muscular surface occurs within approximately three weeks after surgery, as previously demonstrated by video endoscopy [16].

Moreover, the flap’s inset is sutured in a ‘tent’ shape, with slight tension from the posterior edge of the hard palate to the residual tonsillar fossae bilaterally. This configuration fits the defect well and slightly narrows the posterior velopharyngeal space, contributing to better functional outcomes as assessed by speech intelligibility and deglutition (Table 2), which indicates good velopharyngeal function.

In this study, optimal reconstruction of small- to large-sized soft-palate defects was consistently achieved. Most patients reported minimal issues with deglutition, successfully swallowing boluses without difficulty, and maintaining a satisfactory quality of life. These outcomes underscore the effectiveness of the reconstructive methods employed.

Furthermore, the observed correlation between the T stage and speech scores (r = 0.44, *p* = 0.04) aligns with established knowledge, as advanced tumor stages typically result in greater anatomical disruption, negatively affecting speech function [25,26]. Similarly, the trend toward statistical significance between age and deglutition scores (r = 0.37, *p* = 0.09) reflects the expected age-related decline in swallowing efficiency [31]. However, the study’s limited sample size and the subjective nature of the dependent variables likely constrained the ability to identify additional significant relationships. These limitations, combined with variability in individual patient responses, may have obscured weaker yet clinically relevant associations.

Notably, the results demonstrated excellent functional rehabilitation outcomes (Table 3). This success may be attributed to the buccinator muscle flap’s lower propensity for fibrotic retraction compared to skin flaps, with no evidence of shrinkage during the healing process. From our experience, buccinator myomucosal flaps seem to be an ideal option offering several advantages. They allow for the reconstruction of the soft-palate defect using tissue that is the same as or similar to what was lost, in terms of texture, thinness, and mobility, adhering to the principle of “replacing like with like”. Moreover, the flap’s mucosa provides well-vascularized, hairless, and functionally secretive tissue that helps maintain mucosal secretions and sensitivity, which are essential for the rehabilitation of palate functions such as speech articulation and swallowing. Additionally, its muscular component offers the potential for dynamic function. Another advantage of these flaps is that they can be harvested quickly with minimal donor-site morbidity. In all cases, the donor site was repaired using a buccal fat pad taken from the cheek, with no reported donor-site complications (Table 1).

Many efforts have been dedicated to reconstructing soft-palate defects, given its essential role in speech and swallowing. While early approaches included the use of obturators, these prosthetic devices often required significant customization by skilled maxillofacial prosthodontists, leading to patient discomfort and reduced compliance over time [5]. Surgical techniques have since evolved, with options ranging from local and regional flaps to distal free flaps like the radial forearm free flap, which is now the preferred choice for reconstructing large soft-palate defects. The radial forearm flap is particularly valued for its reliability, thinness, pliability, and vascularization, making it well-suited for replacing oropharyngeal mucosa [32]. However, it is associated with high costs, long operating times, and potential donor-site morbidity, which can impact esthetic outcomes. Additionally, the flap’s tendency to shrink during healing, especially after radiotherapy, can impair velopharyngeal function, causing nasal speech and regurgitation.

Among the various reliable flaps described for palate reconstruction, the authors believe that the myomucosal buccinator flap and its tunnelized and islanded variants represent an excellent and relatively easy reconstructive option for post-ablative soft-palate defects.

This study has some limitations, which have to be acknowledged when interpreting the results. First, the retrospective design of our study has possibly led to an inherent selection bias, thus limiting the robustness of our results. Second, this study did not include a control group of patients who underwent alternative surgical techniques, but this had no impact on the main aims of the study. Finally, this study was based on a relatively small sample size; hence, the generalizability to wider populations cannot be confirmed. Despite these limitations, this study has a significant strength as the first to analyze a cohort of patients with a post-resection soft-palate defect, utilizing a comprehensive classification, a myomucosal flap-based reconstructive algorithm, and a complete assessment protocol. Additionally, the results underscore the reliability of known determinants such as tumor stage and patient age, while highlighting the need for larger studies including objective outcome measures to elucidate further the role of defect type and flap reconstruction variables in functional recovery.

## 5. Conclusions

The defect-based classification system introduced here offers a comprehensive, straightforward, and user-friendly method for categorizing soft-palate defects, defining five distinct types. Coupled with a myomucosal reconstructive algorithm, this system is designed to guide surgeons through the reconstructive decision-making process. Its clinical application has demonstrated excellent perioperative and functional outcomes, as reflected in good speech intelligibility, swallowing function, and overall quality of life for patients. The present study is limited by its monocentric design and small sample size, which may have reduced the ability to detect smaller yet clinically meaningful correlations. Future research with larger cohorts is necessary to validate these findings and to explore additional factors influencing postoperative functional recovery, ideally employing objective testing methods. By providing a structured approach to defect assessment and tailored flap reconstruction, this system not only supports better functional rehabilitation but also minimizes donor-site morbidity, making it a valuable tool in advancing head and neck reconstructive surgery.

## Figures and Tables

**Table 1:** Patient demographics, tumors, and treatment details.

Patient No	Sex/Age (Years)	Staging TNM	Type of Defect	Type of Flap	Flap Size (cm) Flap Side	Recipient Site Complications	Donor Site Complications
1	F/58 years	pT2N0MO	Type I	BAMM	4 × 3 homolateral	None	None
2	M/76 years	pT1N0M0	Type II	FAMMIF	6 × 4 homolateral	None	None
3	F/54 years	pT4N2bM0	Type II	t-FAMMIF	7 × 5 contralateral	None	None
4	M/67 years	pT2N0M0	Type IV	t-FAMMIF folded	6 × 3 homolateral	None	None
5	M/54 years	pT2N0M0	Type I	FAMM	6 × 4 homolateral	None	None
6	M/59 years	pT3N0M0	Type V	Kite t-FAMMIF	7 × 5 homolateral	None	None
7	M/65 years	pT1N0M0	Type I	BAMM	4 × 3 homolateral	Minor suture dehiscence	None
8	M/56 years	pT3N0M0	Type III	t-FAMMIF	6 × 4 cm contralateral	None	None
9	M/63 years	pT2N0M0	Type II	FAMMIF	6 × 5 homolateral	None	None
10	M/64 years	pT2N2cM0	Type II	BAMMIF	5 × 4 contralateral	None	None
11	F/58 years	pT2N0M0	Type II	FAMM	6 × 5 homolateral	None	None
12	F/71 years	pT2N0M0	Type IV	t-FAMMIF folded	5 × 5 cm homolateral	None	None
13	M/72 years	pT3N0M0	Type V	Kite t-FAMMIF	7 × 6 cm homolateral	None	None
14	F/75 years	pT3N0M0	Type II	FAMMIF	6 × 5 homolateral	None	None
15	M/54 years	pT2N2cM0	Type II	BAMMIF	6 × 5 homolateral	None	None
16	M/62 years	pT3N2bM0	Type III	t-FAMMIF	6 × 5 cm homolateral	Venous Stasis	None
17	F/70 years	pT3N0M0	Type II	t-FAMM	6 × 5 cm homolateral	Minor suture dehiscence	None
18	M/57 years	pT2N0M0	Type III	t-FAMMIF	7 × 5 homolateral	None	None
19	M/75 years	pT2N0M0	Type IV	t-FAMMIF folded	4 × 4 homolateral	None	None
20	M/83 years	pT3N0M0	Type V	Kite t-FAMMIF	7 × 5 cm homolateral	None	None
21	M/76 years	pT1N0M0	Type I	BAMM	4 × 4 cm homolateral	None	None
22	M/70 years	pT3N0M0	Type V	Kite t-FAMMIF	7 × 5 contralateral	None	None

**Table 2:** Postoperative functional assessment.

Patient No	Sex/Age (Years)	Deglutition Score # (Score 0–7)	Speech Score * (Score 0–4)
1	F/58 years	6	0
2	M/76 years	7	0
3	F/54 years	5	1
4	M/67 years	7	0
5	M/54 years	5	1
6	M/59 years	7	0
7	M/65 years	6	0
8	M/56 years	5	1
9	M/63 years	6	0
10	M/64 years	7	0
11	F/58 years	6	1
12	F/71 years	5	1
13	M/72 years	7	0
14	F/75 years	7	0
15	M/54 years	6	0
16	M/62 years	6	0
17	F/70 years	4	2
18	M/57 years	6	0
19	M/75 years	5	1
20	M/83 years	7	0
21	M/76 years	6	0
22	M/70 years	6	0
Total (mean ± SD)		**6.04 ± 0.85**	**0.36 ± 0.58**

# Deglutition assessment: the score ranges from 1 (severe complaints and unable to swallow) to 7 (no complaints). Speech score * was assessed by VPF: (0): competent, (1): borderline competent, (2): mild-to-moderate VPI, and (3): severe VPI.

**Table 3:** Quality of life assessment.

Patient No	Sex/Age (Years)	Physical Well-Being (Range 0–28)	Social/Family Well-Being (Range 0–28)	Emotional Well-Being (Range 0–24)	Functional Well-Being (Range 0–28)	H&N Cancer Subscale (Range 0–40)
1	F/58 years	27	28	21	26	40
2	M/76 years	28	25	21	25	40
3	F/54 years	23	10	8	18	34
4	M/67 years	27	28	24	28	40
5	M/54 years	28	28	24	27	38
6	M/59 years	26	28	24	27	40
7	M/65 years	15	18	16	22	33
8	M/56 years	20	26	18	20	34
9	M/63 years	26	25	21	25	39
10	M/64 years	25	27	23	26	38
11	F/58 years	24	27	23	27	38
12	F/71 years	26	28	22	26	38
13	M/72 years	27	28	23	28	40
14	F/75 years	27	28	21	26	39
15	M/54 years	26	25	15	27	33
16	M/62 years	23	25	11	24	34
17	F/70 years	20	21	9	17	35
18	M/57 years	24	21	16	20	34
19	M/75 years	20	21	15	18	33
20	M/83 years	27	26	21	17	38
21	M/76 years	26	25	21	22	39
22	M/70 years	24	21	16	22	38
Total (mean ± SD)		**24.5 ± 3.26**	**24.5 ± 4.39**	**18.7 ± 4.87**	**23.5 ± 3.66**	**37.04 ± 2.61**

Functional Assessment of Cancer Therapy—Head and Neck Questionnaire.

**Table 4:** Correlation analysis between clinical parameters and functional outcomes. Significant Spearman’s correlations (*p* < 0.05) have been highlighted in bold.

Independent Variable	Dependent Variables	Correlation (r)	*p*-Value
Age	● Deglutition Score ● Speech Score ● Physical Well-being ● Social Well-being ● Emotional Well-being ● Functional Well-being ● H&N Cancer Subscale	0.37, −0.23, 0.23, 0.03, 0.09, −0.20, 0.34	0.09, 0.30, 0.31, 0.88, 0.68, 0.37, 0.12
T Stage		−0.33, 0.44, −0.21, −0.08, −0.18, −0.29, −0.27	0.13, **0.04**, 0.35, 0.72, 0.43, 0.19, 0.23
Type of Defect		0.16, 0.03, −0.08, 0.07, 0.02, −0.10, 0.08	0.48, 0.89, 0.71, 0.74, 0.92, 0.67, 0.73
Flap Area		0.17, −0.06, 0.04, −0.06, −0.09 , −0.04, 0.03	0.45, 0.78, 0.86, 0.79, 0.67, 0.88, 0.88

## Data Availability

The data presented in this study are available on request from the corresponding author to allow reproducibility of results.
